# Mesoscale calcium imaging *in vivo:* evolution and contribution to developmental neuroscience

**DOI:** 10.3389/fnins.2023.1210199

**Published:** 2023-08-01

**Authors:** Teresa Guillamón-Vivancos, Dorien Vandael, Daniel Torres, Guillermina López-Bendito, Francisco J. Martini

**Affiliations:** Instituto de Neurociencias de Alicante, Universidad Miguel Hernández-Consejo Superior de Investigaciones Científicas (UMH-CSIC), Sant Joan d’Alacant, Spain

**Keywords:** mesoscale imaging, *in vivo* calcium imaging, neural development, circuit development, mouse

## Abstract

Calcium imaging is commonly used to visualize neural activity *in vivo*. In particular, mesoscale calcium imaging provides large fields of view, allowing for the simultaneous interrogation of neuron ensembles across the neuraxis. In the field of Developmental Neuroscience, mesoscopic imaging has recently yielded intriguing results that have shed new light on the ontogenesis of neural circuits from the first stages of life. We summarize here the technical approaches, basic notions for data analysis and the main findings provided by this technique in the last few years, with a focus on brain development in mouse models. As new tools develop to optimize calcium imaging *in vivo*, basic principles of neural development should be revised from a mesoscale perspective, that is, taking into account widespread activation of neuronal ensembles across the brain. In the future, combining mesoscale imaging of the dorsal surface of the brain with imaging of deep structures would ensure a more complete understanding of the construction of circuits. Moreover, the combination of mesoscale calcium imaging with other tools, like electrophysiology or high-resolution microscopy, will make up for the spatial and temporal limitations of this technique.

## Introduction

The complex functions of the brain rely on a communication system that is based on the electrical and chemical messages used by neurons to transmit information. Since Ramon y Cajal formulated the neuron doctrine, neuroscientists have tried to understand, and visualize, this communication system in action. To assess cell-to-cell communication in the nervous system, there is currently an extensive repertoire of experimental approaches mostly based on electrophysiological and optical techniques. Developed by the end of the twentieth century (Tsien, 1980), the methods based on imaging of intracellular calcium levels to infer neuronal activity have meant a paradigm shift in the field of neuroscience.

Cells keep their cytosolic calcium concentration at low levels (~100 nM). To do this, they employ an heterogenous toolkit of calcium-binding molecules and multiple membrane channels, exchangers and pumps located at the cell and organelles surface (Berridge, 1998; Berridge et al., 2003; Clapham, 2007). Although a steady high calcium is cytotoxic (Orrenius et al., 2003), calcium concentration is not invariably low. Cells undergo sudden and transient calcium elevations that serve as an intracellular signal to induce neural transmission or processes such as phosphorylation and transcription. In this way, intracellular calcium controls different effector mechanism that determine the physiology of the cell. In particular, during brain development, intracellular calcium signaling mediates multiple functions including neuronal induction, proliferation, migration and differentiation (Uhlén et al., 2015; Toth et al., 2016; Arjun McKinney et al., 2022). Calcium ions flux into the cytoplasm released from the internal stores or transported from the extracellular fluid. Calcium influx in developing neurons occurs through multiple pathways, implicating voltage-, ligand-, store-, mechanical-, and receptor-operated channels (Uhlén et al., 2015; Toth et al., 2016; Arjun McKinney et al., 2022).

Calcium sensors are fluorescent molecules that allow for optical measurement of the levels of cytosolic calcium. After binding calcium ions, sensors undergo a shift in the quantum yield of fluorescence or in the emission/excitation wavelength. Nowadays, some of the most commonly used calcium sensors are the GCaMP proteins, a family of genetic fluorescent indicators based on the combination of a green fluorescent protein and a calcium binding protein named calmodulin (Miyawaki et al., 1997; Nakai et al., 2001; Tian et al., 2009; Akerboom et al., 2012; Chen et al., 2013; Zhang et al., 2023). The development of these and other calcium sensors caused a paradigm shift in the field of Systems and Developmental Neuroscience. For example, calcium imaging in slices provided the first evidence of cell assemblies of synchronically active neurons in the developing cortex (Yuste et al., 1992). These neuronal domains are the developmental basis of functional cortical columns and pharmacological experiments show that they are connected by gap junctions (Yuste et al., 1995; Dupont et al., 2006). In slices, these synchronous events of activity have been shown to propagate across large distances, for instance, from the septum or the ventral cortex to the dorsal cortex. Importantly, these studies show that the patterns of propagation and the neurotransmitters that mediate these waves change during development (Conhaim et al., 2011; Easton et al., 2014).

Calcium imaging *in vivo* in rodents further confirmed the presence of neuronal domains of activity during cortical development. These domains emerge both spontaneously or evoked by peripheral stimulation (Kummer et al., 2016; Nakazawa et al., 2020), and undergo a slight reduction in size during the first postnatal week (Yang et al., 2013; Nakazawa et al., 2020). The development of powerful high-resolution imaging techniques allowed calcium imaging at cellular and subcellular resolution level, evidencing developmental changes in dendrites, spines or axonal branching (Lendvai et al., 2000; Portera-Cailliau et al., 2005; Zuo et al., 2005; Carrillo et al., 2013; Nakazawa et al., 2018).

Wide-field imaging *in vivo* offers the possibility of sampling large fields of view comprising multiple brain areas or even the whole dorsal surface of the brain in mice. As large as millimetres or centimetres, fields of view exceed the standard microscopic scale and, therefore, this approach is referred to as mesoscale imaging. Wide-field mesoscale imaging provides enough spatiotemporal resolution to capture supracellular domains of calcium changes occurring throughout the mouse cortex, superior and inferior colliculus. In Developmental Neuroscience, imaging a large extent of the neuraxis allows interrogation of distributed immature networks in the brain and gave rise to ground-breaking findings such us the simultaneous recording of retinal waves in the cortex and superior colliculus (Ackman et al., 2012) or the developmental progression of the segregation of multimodal responses in sensory pathways (Guillamón-Vivancos et al., 2022).

Although neuronal activity can be imaged at the mesoscopic level using dyes or blood flow-related optical techniques (Orbach et al., 1985; Grinvald and Hildesheim, 2004; Berwick et al., 2005; Ma et al., 2016b; Mateo et al., 2017), we will focus in this review on techniques and findings obtained from brains of mouse models that carry genetically encoded calcium indicators (GECIs). The mouse brain has been the most recurrent subject studied under this approach due to its small size and the relatively standard manipulations available. In particular, we will describe how this technique has contributed to elucidate fundamental processes of circuit construction and maturation during brain development.

## Technical approaches to mesoscopic imaging of the developing brain *in vivo*

**Figure 1:**
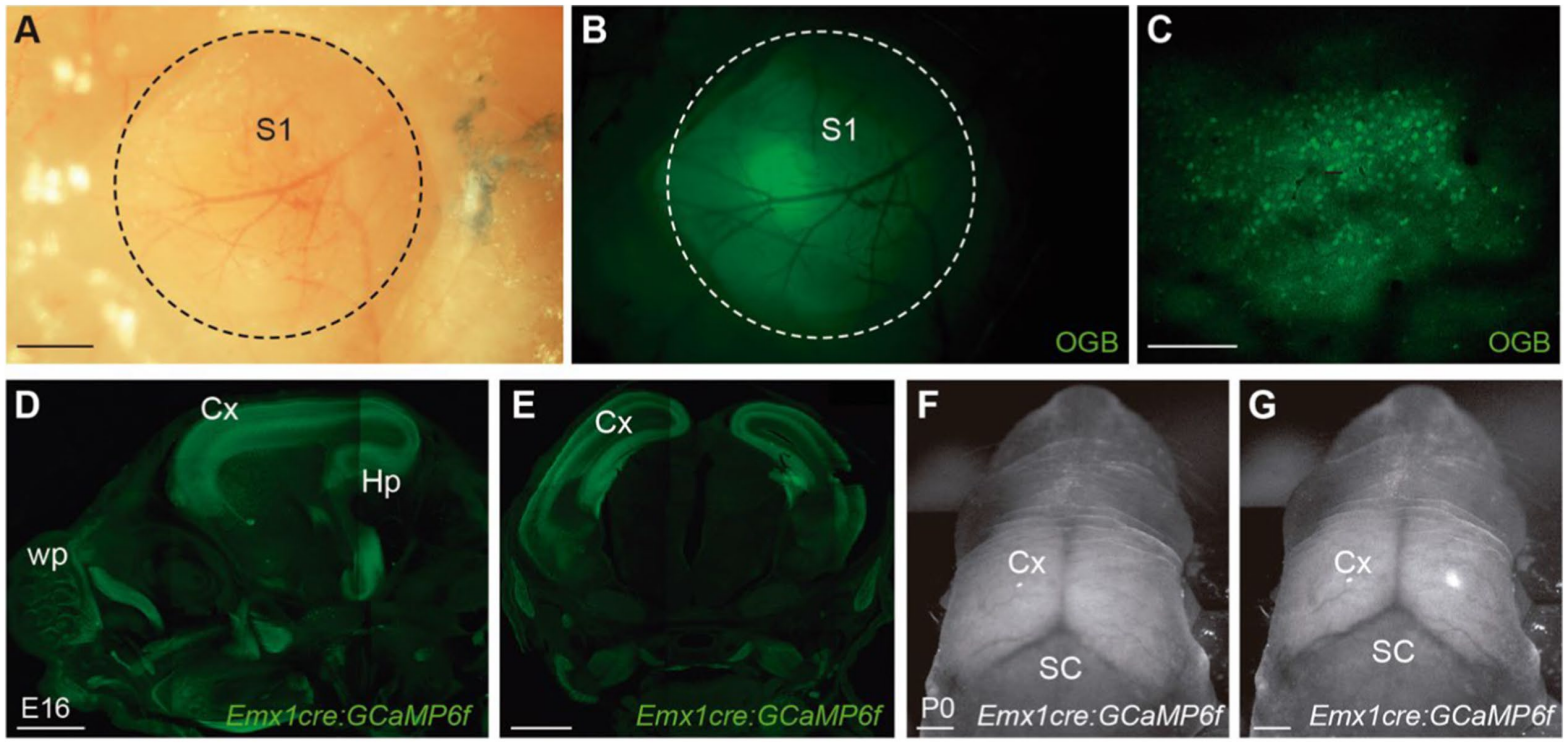
Transgenic mice allow for brain-wide and cell-specific expression of calcium indicators. **(A,B)** Wide-field dorsal view of S1 in an adult mouse injected with the calcium dye Oregon Green 488 BAPTA AM (OGB) through a 2 mm-craniotomy (circle). **(C)** Two-photon image of layer 2/3 cells loaded with OGB, 1 h after injecting the dye. **(D)** Sagittal and **(E)** coronal section of a transgenic mouse that expresses GCaMP6f in all excitatory cortical neurons under the Emx1 promoter. **(F,G)** Wide-field image of a Emx1cre:GCaMP6f P0 mouse showing baseline fluorescence **(F)** and a cortical calcium event **(G)**. Scale bars: 500 μm in **(A,B)**, 100 μm in C, 1000 μm in **(D–G)**. Panels **(A–C)** courtesy of Dr. M. Maravall, University of Sussex, Brighton, UK.

*In vivo* calcium imaging requires the appropriate filling of neuronal cells with fluorescent calcium indicators. These calcium indicators can be divided into two major groups: chemically engineered (Tsien, 1988, 1989a,b) and genetically encoded indicators (Miyawaki et al., 1997, 1999). Chemically engineered calcium indicators were first developed more than 30 years ago by Tsien (1988). These synthetic dyes are loaded *in vivo* via incubation or bolus injections into the brain (Sobel and Tank, 1994; Svoboda et al., 1999; Stosiek et al., 2003; Garaschuk et al., 2006) (Figures 1A–C). More recently, the field shifted almost completely to the use of GECIs. The DNA coding for the calcium indicator protein is delivered into the cells of interest by electroporation, injection of viral vectors or through the generation of transgenic mouse lines. Compared to synthetic dyes, GECIs allow for targeting specific cell-types (Figures 1D–G) or subcellular compartments. In addition, their expression is stable over time, which enables to conduct long-term experiments (Mostany et al., 2013).

Recombinant adeno-associated viruses (AAVs) are commonly used as vectors for *in vivo* gene delivery (Foust et al., 2009). Viral vectors, encoding GECIs, can be delivered by intracerebral (Goldey et al., 2014) or intraventricular injections (Kim et al., 2014). However, these methods often lead to highly variable expression patterns due to different virus concentrations and are associated with cell damage or death (Grødem et al., 2023). Recently, whole brain-gene delivery was achieved by retro-orbital injections of AAV9 and other engineered AAV variants (Chan et al., 2017). However, the major drawback of this approach is that robust expression takes several weeks after the time of injection (Chan et al., 2017), which is a severe limitation for the study of the developing mouse brain. Since AAV9 particles cross the blood–brain barrier, intravenous injection through the tail or temporal vein may be used for massive delivery of viruses to achieve broad expression levels across the brain. This strategy, however, entails many disadvantages such as large injection volumes, high financial cost, tissue damage, and suboptimal transduction efficiency (Foust et al., 2009; Kim et al., 2013). Another strategy to massively deliver the virus into the brain is through injections into the transverse sinus. In mouse neonates, these injections can be performed directly through their thin scalp and skull. When the virus is injected as early as postnatal day (P) 0, the calcium indicator reaches a dense and stable expression already by P4 (Hamodi et al., 2020), which is faster than intravenous gene delivery.

To avoid invasive techniques and inhomogeneous expression, neuroscientists have developed transgenic mice that express genes coding for calcium sensors (Zariwala et al., 2012; Dana et al., 2014). Moreover, further development of specific transgenic mouse lines that only target a subpopulation of neurons allows discrimination of activity from different cell types, regions, or sub-regions such as cortical layers (Taniguchi et al., 2011; Madisen et al., 2015). It is also possible to label two different subpopulations in the same mouse line using green and red fluorescent calcium indicators allowing for simultaneous imaging of different neuronal subpopulations (Dana et al., 2016; Wei, 2019; Cardin et al., 2020).

**Figure 2:**
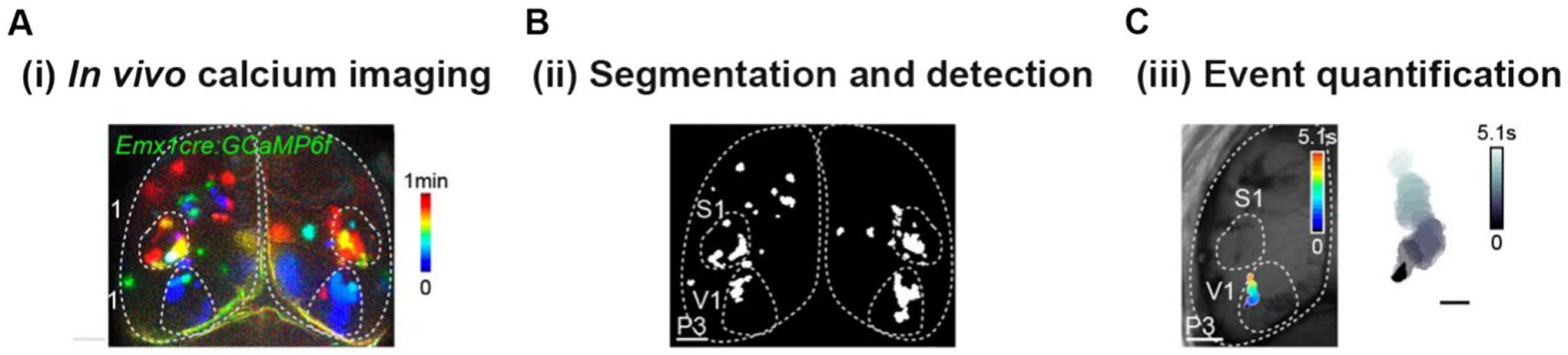
Essential pipeline for data acquisition and analysis of wide-field fluorescence calcium imaging. **(A)** Temporal color-coded projection of a one-minute recording of spontaneous cortical activity in a P3 awake mouse. **(B)** Image segmentation of the movie shown in **(A)** using intensity thresholds (detected domains in white). **(C)** Activity domains are detected as solid objects whose shape changes over time. Scale bar: 1000 μm.

Overall, transgenic mice that express GECIs have emerged as a reliable tool for recording activity *in vivo* across the neocortex. Different reports, however, have raised some concerns by demonstrating aberrant activity in high-level transgene expression lines. For instance, epileptiform activity was observed when Emx1CRE mice were crossed with mouse lines Ai93 and Ai94, expressing GCaMP6 both under CRE and the tetracycline-controlled transactivator (tTA) conditions (Steinmetz et al., 2017; Daigle et al., 2018). This effect seems to be related to the very high levels of tTA gene expression in early nervous system development (Daigle et al., 2018). By using Emx1CRE mouse lines that express GCaMP6 only under the regulation of the Cre recombinase, like Ai95 (Figures 1D–G, 2) and Ai96, epileptiform events do not occur (Steinmetz et al., 2017). Another point to keep in mind is that calcium sensors have buffering actions, which will reduce the amplitude of intracellular calcium changes and will affect the decay time of the fluorescent signal (Neher, 1995; Higley and Sabatini, 2008; Grienberger and Konnerth, 2012). Recognizing the impact and artefacts of the sensors on calcium signaling will allow investigators to use these tools more effectively (McMahon and Jackson, 2018). To conclude, some forethought is required when using GECIs lines that drive the broad expression of calcium sensors throughout the nervous system and early in development.

The *in vivo* preparation for calcium imaging in developing mice is always technically challenging. The handling and manipulation of perinatal mice requires different strategies and skills from those used for adult animals. For instance, at early postnatal stages (P0 and P1), pups are only viable and healthy without the mother for a limited time (~2 h), as long as their temperature is strictly controlled. In addition, since the skin and skull are sufficiently transparent it is possible to perform wide-field imaging through them (Antón-Bolaños et al., 2019; Guillamón-Vivancos et al., 2022). However, in this scenario, a strong fluorescence signal is needed and the contribution of the skin and skull to light scattering must be taken into account (Waters, 2020). Furthermore, although the perinatal preparation does not need intensive surgeries and controlled aftercare of pups, the frailness of the tissue hampers head fixation and stabilization, especially during body movements (Blumberg et al., 2015). Imaging mouse embryos is even more challenging than early postnatal pups. However, different reports have recently described methods that implement wide-field cortical imaging in preterm stages (Antón-Bolaños et al., 2019; Guillamón-Vivancos et al., 2022). And, additionally, a para-uterine method has been developed that provides enhanced mechanical stability to the embryo for the acquisition of 2-photon imaging in combination with electrophysiological recordings (Munz et al., 2023).

## Data acquisition and analysis of multi-region neuronal activity

Wide-field imaging on perinatal mice imposes some special considerations regarding image acquisition due to the specimen size and its fragility. In addition, the expression time of GECIs could be longer than the short temporal window available to conduct perinatal studies. Low expression levels derive from either the dynamics of viral vectors or the temporal profile of the promoter controlling the reporter in transgenic mice (Zhang et al., 2011a). Also, since it is convenient to preserve the delicate scalp and skull as mentioned above, scattering likely increases the degradation of the fluorescent signal (Hillman et al., 2011; Waters, 2020). On the other hand, the spatiotemporal features of neuronal activity in perinatal cortices facilitates their detection at the mesoscale level. Activity in immature cortical circuits emerges as correlated events of neighboring neurons that generates calcium domains of dozen to hundreds of microns in diameter. This pattern becomes progressively more sparse with further maturation of the cortex (Nakazawa and Iwasato, 2021; Weiser et al., 2023). Mesoscale imaging enables the acquisition of calcium domains using large fields of view (~10 to 500 mm^2^) and during several minutes. This is especially relevant when studying the progression of the patterns of spontaneous activity and the extent of correlation among different cortical regions. Spatial resolution values usually range from tens to hundreds of microns per pixel (supra-cellular resolution), whereas the typical frame rates span from 3 to 30 Hz with common values around 5 to 10 Hz (Hillman et al., 2011; Zhang et al., 2011b; Ackman et al., 2012, 2014; Xu et al., 2015; Babola et al., 2018; Gribizis et al., 2019; Ge et al., 2021; Ren and Komiyama, 2021; Wang et al., 2021). Overall, researchers configure the acquisition settings based on their specific scientific question and on empirical facts such as the intensity of the signal. However, the field needs more technical reports studying the balance between acquisition settings and signal quality in order to generate robust datasets to feed analysis workflows (Vanni et al., 2017; Weiser et al., 2023).

The raw wide-field signal encloses confounding factors that represent a challenge for image processing and data interpretation. Confounding factors have been mostly studied in adult brains and include scattering, bleaching, contamination with background signal in the absence illumination, autofluorescence and hemodynamic absorption, and motion of the sample due to body movements, heartbeat, or breathing (Hillman et al., 2011; Ma et al., 2016a). Bleaching, slow baseline fluctuations and background signal are usually corrected using algorithms that substract them from the raw signal. Motion artifacts in the horizontal plane are often minimized making use of alignment algorithms or discarding movement epochs (Ackman et al., 2014; Saxena et al., 2020; Wang et al., 2021). Conversely, some other factors, such as autofluorescence, are not strong enough to distort the signal and they are usually ignored (Kozberg and Hillman, 2016; Ma et al., 2016a). Lastly, the need for hemodynamic corrections is not clear and requires special attention as neurovascular coupling changes throughout development (Kozberg and Hillman, 2016).

Researchers have developed analysis routines to pre-process, detect and quantify mesoscopic calcium signals. Pre-processing includes removing bleaching, background, and artifacts, sharpening the neural signal, and computing ratiometric measurements (like deltaF/F_0_). Although a standard and consolidated toolbox is missing, the typical workflow after pre-processing is based on methods that segment digital images using intensity thresholds and detect activity domains as 2D solid objects whose shape varies over time (Figure 2). After detection, there are many features that can be quantified from mesoscopic activity events. Basic features include the occurrence, duration, and spatial and morphological metrics of events (Ackman et al., 2014; Ma et al., 2016a). Additionally, more sophisticated analytical tools use optical flow measures to characterize the sources and sinks of neural activity, direction, trajectory, and speed of propagation for each event (Afrashteh et al., 2017; Linden et al., 2021).

Mesoscale approaches allow for simultaneous recording of the whole or a large portion of the brain dorsal surface. Therefore, assisted by a reference atlas, the functional interareal connectivity could be calculated based on correlations among active areas (White et al., 2011; Mohajerani et al., 2013; Chan et al., 2015; Silasi et al., 2016). In the developing brain, since anatomical maps are rapidly changing, it is convenient to use data-driven approaches whereby the cortex is parcellated according to the information extracted from the spontaneous activity by matrix factorization methods (i.e., PCA and ICA) (Makino et al., 2017; Saxena et al., 2020; Couto et al., 2021; Weiser et al., 2023). In either case, to corroborate that found parcels actually correspond to developing brain regions, we suggest to provide further confirmation using anatomical tracings or genetic data.

While there are analytical toolboxes available online or at repositories such as GitHub (Table 1),^1^ they are usually tailored to address context-specific questions using lab-specific datasets, often obtained from adult samples. In the near future, it would be helpful to rely on a standard toolbox that enables straightforward comparison among different datasets.

**Table 1:** Summary of toolboxes available online for calcium image analysis.

Name	Website	Method	Reference
wholeBrainDX	https://github.com/ackman678/wholeBrainDX	Based on image processing: thresholding techniques for movie segmentation followed by the characterization of calcium objects detected in the movie (area, duration, shape, etc.).	Ackman et al. (2014)
CrairLabCode	https://github.com/agribizis/CrairLabCode	Based on image processing: thresholding techniques for movie segmentation followed by the characterization of calcium objects detected in the movie (area, duration, shape, etc.).	Gribizis et al. (2019)
Yixiang_OOP_pipeline	https://github.com/CrairLab/Yixiang_OOP_pipeline	Based on image processing: thresholding techniques for movie segmentation followed by the characterization of calcium objects detected in the movie (area, duration, shape, etc.).	Wang et al. (2021)
OFAMM	https://github.com/navvab-afrashteh/OFAMM	Based on optical flow methods from computer vision to characterize activity in terms of sources, sinks, and trajectories in between.	Afrashteh et al. (2017)
FLOWPortrait	https://github.com/natejlinden/FLOWPortrait	Based on optical flow methods from computer vision to characterize activity in terms of sources, sinks, and trajectories in between.	Linden et al. (2021)
Wide-field-calcium-imaging	https://github.com/CRen2333/Wide-field-calcium-imaging	Based on matrix factorization techniques: PCA-ICA for movie segmentation followed by the exploration of the components.	Ren and Komiyama, 2021
pySEAS	https://github.com/ackmanlab/pySEAS	Based on matrix factorization techniques: PCA-ICA for movie segmentation followed by the exploration of the components.	Weiser et al. 2023
wfield	https://github.com/churchlandlab/wfield	Based on matrix factorization techniques: NMF for movie segmentation followed by the exploration of the components.	Couto et al. (2021)

## Recent advances in developmental neuroscience yielded by mesoscale calcium imaging

Due to the straightforward stimulation and manipulation of sensory organs, together with the fact that sensory cortices are readily accessible from a birds-eye view, some of the most relevant insights into Developmental Neuroscience yielded by mesoscopic imaging have to do with sensory systems.

In the visual system, for instance, mesoscopic imaging has been used to describe the spatiotemporal dynamics of retinal waves that propagate directly to the superior colliculus and to the cortex via the visual thalamus. Retinal waves originate in the developing retina and can be subdivided into stages I to III, depending on the cell-types involved and the mode of neurotransmission. Stage II retinal waves, for instance, starting at around P1 until P9 in mice, have been shown to have a role in the refinement of retinotopic maps in the superior colliculus and the primary visual cortex (Ackman et al., 2012; Burbridge et al., 2014; Gribizis et al., 2019). As opposed to peripherally-generated activity, cortical activity generated by local cortico-cortical connections seems to be involved in the generation of “large-scale” modules of activity in the visual cortex that are the basis of orientation columns (Smith et al., 2018), as shown in ferrets. By combining functional correlation analyses of spontaneous activity in the visual cortex with anatomical tracings in P5 to P14 mice, Murakami et al. (2022) demonstrated that connectivity modules from subcortical regions to primary visual and higher visual areas develop in parallel.

Simultaneous mesoscopic imaging of the inferior colliculus and the auditory cortex has provided important insights into the development of the auditory system as well. For example, Babola et al. (2018) demonstrated that there are correlated patterns of activity in these areas during development that match future tonotopic maps. These bursts of activity propagate from the developing cochlea before hearing onset and depend on purinergic signaling (Babola et al., 2021). Interestingly, the strongest of these bursts of neuronal activity in the inferior colliculus also trigger large-scale coordinated calcium waves in astrocytes, and these coincident astrocyte-neuron events are restricted to early development (Kellner et al., 2021). Removing spontaneous activity in the cochlea reduces the frequency of spontaneous activity in the inferior colliculus but increases its area of activation, resulting in neurons with increased gain and broader frequency sensitivity (broadening of receptive fields) when they are ready to respond to auditory stimuli; additionally, the area of the cortex dedicated to process tonal information becomes compressed (Kersbergen et al., 2022).

Beyond spontaneous activity, mesoscale imaging can be used to record cortical activity in response to a peripheral stimulus. Antón-Bolaños et al. (2019) demonstrated that a peripheral tactile stimulus at embryonic stages in mice triggers a neural response in the corresponding cortical area. This means that, already during embryonic life, the somatosensory system has developed the capability to transfer an external stimulus, through the multiple subcortical stations of the somatosensory system, all the way to the cortex.

Mesoscale imaging allows for simultaneous recording of activity from both hemispheres or from brain areas which could be separated long distances in the neuraxis. Combining this advantage with peripheral stimulation and pharmacological interventions, it was recently demonstrated that somatosensory and visual modalities emerge intermingled at embryonic stages and only become independent at birth, when the earliest form of retinal activity (stage I waves) arrives at the superior colliculus (Guillamón-Vivancos et al., 2022).

To understand how the patterns of spontaneous activity affect brain development, it is necessary to comprehend the variables that modulate these patterns. One important factor that affects spontaneous activity is the animal’s behavioral state represented by sleep–wake cycles. In mice, behavioral states are present from birth (Blumberg et al., 2020) and they are inferred at neonatal stages mostly from muscle tone and body movements (Seelke and Blumberg, 2008). At these stages, EEG is an unreliable tool for sleep scoring because early electrical signatures change rapidly across development and do not resemble adult patterns (Blumberg et al., 2020). Using mesoscale calcium imaging while monitoring behavioral state, it has been shown that large events of spontaneous activity are confined to sleep periods by the end of the first postnatal week in mouse pups (Mojtahedi et al., 2021; Tabuena et al., 2022). At earlier stages, the frequency of large events is independent of sleep–wake states. However, they show a state-specific spatial preference whereby significantly more events are detected towards the somatosensory and motors cortices during periods of body movement. Conversely, activity is preferentially detected in the occipital regions during resting periods (Mojtahedi et al., 2021; Tabuena et al., 2022). In sum, behavioral state influences the kind of activity observed in the cortex and, thus, it should be taken into account when conducting experiments using perinatal mice.

## Future perspectives

Mesoscale imaging of neural activity would be of particular interest to understand the emergence of circuits involved in behaviors and higher order functions like memory, attention, cognition, as well as resting-state spontaneous patterns (Matsui et al., 2016; Vanni et al., 2017; Wright et al., 2017). Recently, combining wide-field imaging with optogenetics and a visual working memory task, Voitov and Mrsic-Flogel determined that working memory is maintained by activity loops distributed throughout the cortex (Voitov and Mrsic-Flogel, 2022). The understanding of how these and other distributed networks emerge during development is currently missing and wide-field imaging tools seem one of the best approaches to start shedding light on this topic. Mesoscopic imaging might also be applied to understand how the brain behaves under pathological conditions. For instance, in adult mice, neural activity has been shown to be altered by seizures (Rossi et al., 2017; Montgomery et al., 2020). Future studies will require to use wide-field imaging to understand circuit reorganizations in neurodevelopmental disorders. By linking the information of mesoscopic imaging to genetic mutations, environmental influences or behavior, this tool could have translational applications by anticipating diseases in later stages of life.

Because of the low spatial resolution and limited imaging depth of mesoscale approaches, it would be particularly interesting to combine this tool with other techniques that can make up for these limitations, like electrophysiology, fMRI or 2-photon microscopy (Clancy et al., 2019; Peters et al., 2021; Barson et al., 2020; Lake et al., 2020). Although wide-field imaging has been mostly performed in readily accessible brain structures, like the dorsal cortex and midbrain, it is imperative to develop new preparations that allow for imaging of structures sitting deeper in the brain. In combination with imaging of cortical structures, gaining access to deep structures, like the thalamus (Murakami et al., 2022), will provide a more complete understanding of the whole circuitry. Furthermore, a combination of mesoscopic imaging with selective manipulations through optogenetics (Voitov and Mrsic-Flogel, 2022) in the developing brain may allow for a detailed understanding of the formation of specific circuits. Additionally, the study of activity in slices that preserve specific connections might help bridge the gap between mesoscale findings and local circuitry underlying them (Mukherjee et al., 2023). This multidimensional analysis will provide unique insights into the building of circuits during brain development.

## Author contributions

All authors listed have made a substantial, direct, and intellectual contribution to the work and approved it for publication.

## Funding

This work was supported by grants from the European Research Council (ERC-2021-ADG-101054313) Generalitat Valenciana (PROMETEO/2021/052) Spanish Ministry of Science, Innovation and Universities (grants: FJC2021-046529-I and PID2021-127112NB-I00). “La Caixa” Foundation (Health Research 2020/HR20-00083).

## Conflict of interest

The authors declare that the research was conducted in the absence of any commercial or financial relationships that could be construed as a potential conflict of interest.

## Publisher’s note

All claims expressed in this article are solely those of the authors and do not necessarily represent those of their affiliated organizations, or those of the publisher, the editors and the reviewers. Any product that may be evaluated in this article, or claim that may be made by its manufacturer, is not guaranteed or endorsed by the publisher.

## Footnotes

^1^http://neurocaas.org/
